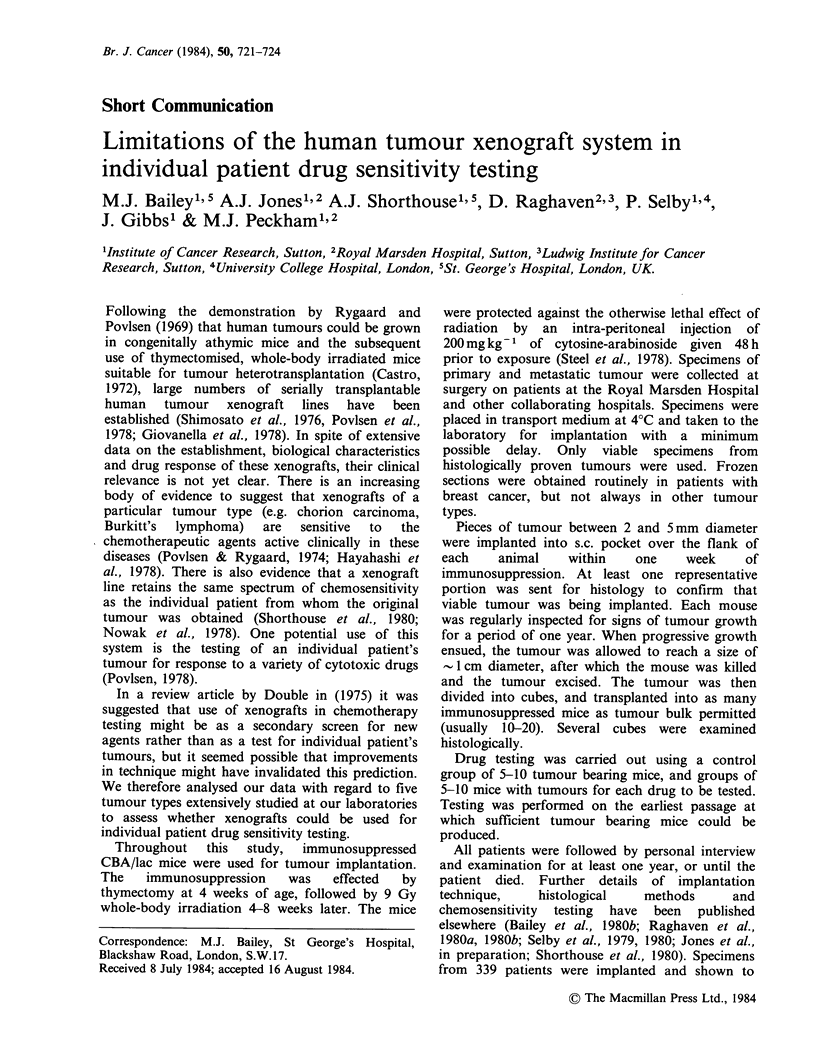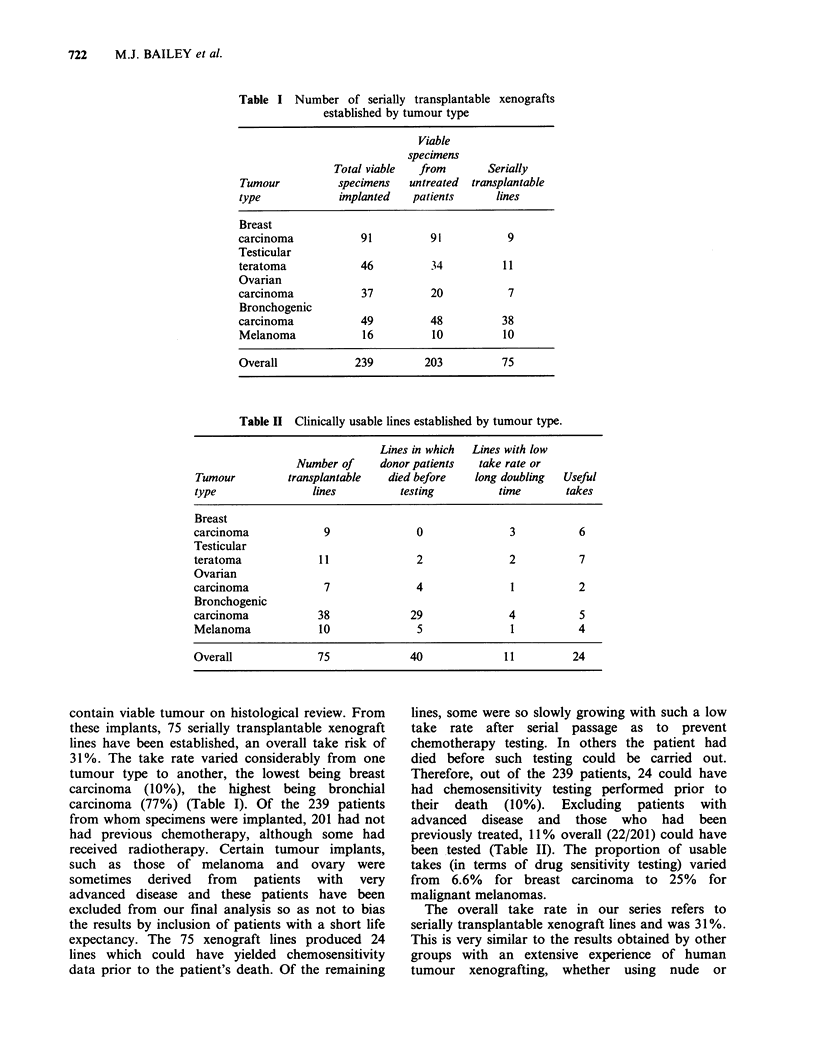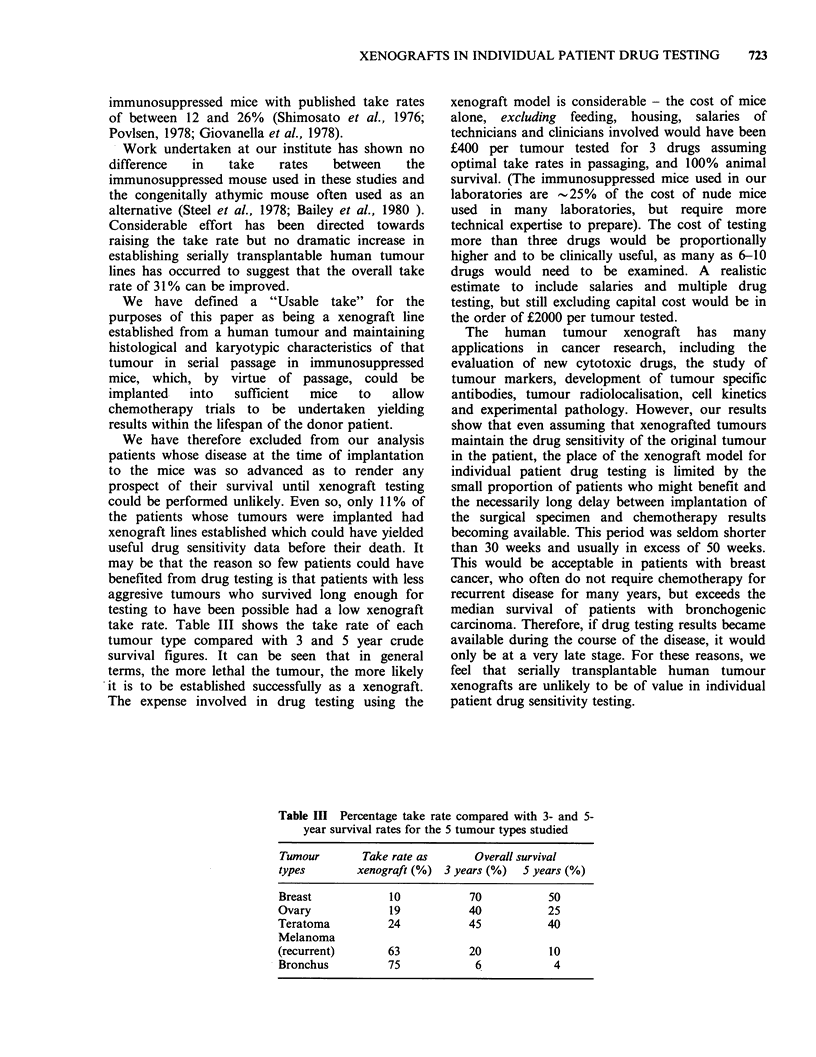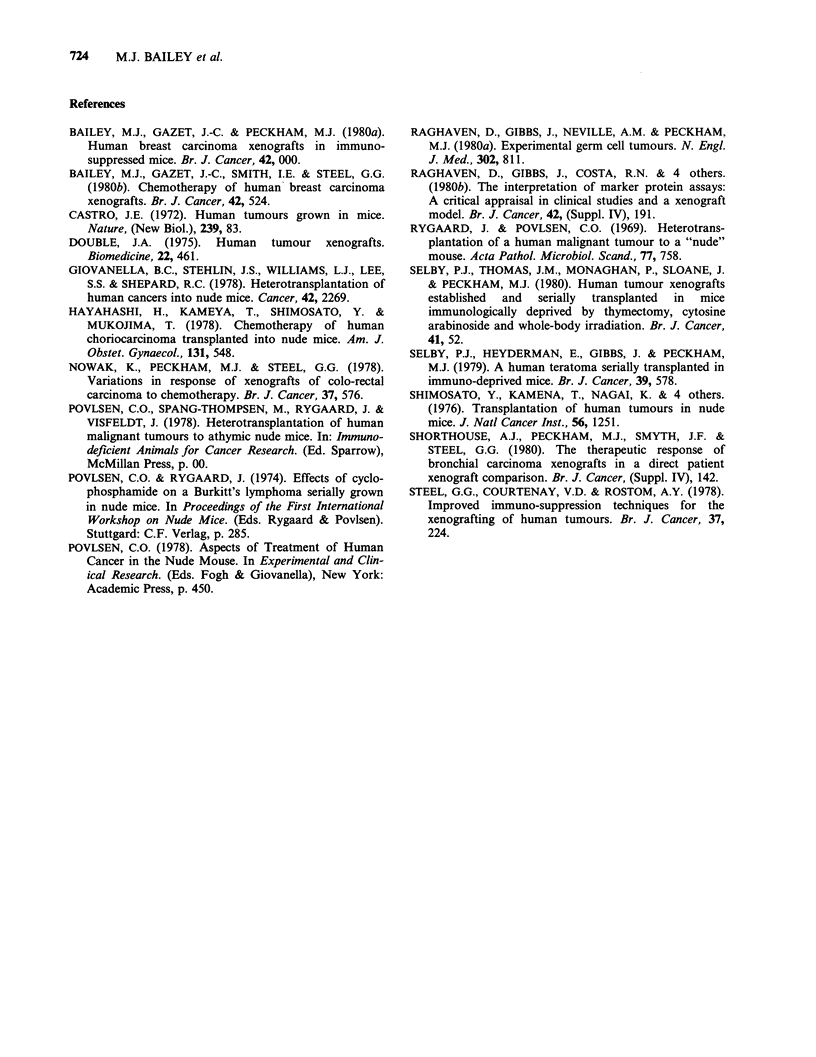# Limitations of the human tumour xenograft system in individual patient drug sensitivity testing.

**DOI:** 10.1038/bjc.1984.242

**Published:** 1984-11

**Authors:** M. J. Bailey, A. J. Jones, A. J. Shorthouse, D. Raghaven, P. Selby, J. Gibbs, M. J. Peckham


*Br. J. Cancer* (1984), **50**, 721–724

### Short Communication

### Limitations of the human tumour xenograft system in individual patient drug sensitivity testing

M.J. Bailey[1,5] A.J. Jones[1,2] A.J. Shorthouse[1,5], D. Raghaven[2,3], P. Selby[1,4], J. Gibbs[1] & M.J. Peckham[1,2]

[1]*Institute of Cancer Research, Sutton,* [2]*Royal Marsden Hospital, Sutton,* [3]*Ludwig Institute for Cancer Research, Sutton,* [4]*University College Hospital, London,* [5]*St. George's Hospital, London, UK.*

Following the demonstration by Rygaard and Povlsen (1969) that human tumours could be grown in congenitally athymic mice and the subsequent use of thymectomised, whole-body irradiated mice suitable for tumour heterotransplantation (Castro, 1972), large numbers of serially transplantable human tumour xenograft lines have been established (Shimosato *et al.*, 1976, Povlsen *et al.*, 1978; Giovanella *et al.*, 1978). In spite of extensive data on the establishment, biological characteristics and drug response of these xenografts, their clinical relevance is not yet clear. There is an increasing body of evidence to suggest that xenografts of a particular tumour type (e.g. chorion carcinoma, Burkitt's lymphoma) are sensitive to the chemotherapeutic agents active clinically in these diseases (Povlsen & Rygaard, 1974; Hayahashi *et al.*, 1978). There is also evidence that a xenograft line retains the same spectrum of chemosensitivity as the individual patient from whom the original tumour was obtained (Shorthouse *et al.*, 1980; Nowak *et al.*, 1978). One potential use of this system is the testing of an individual patient's tumour for response to a variety of cytotoxic drugs (Povlsen, 1978).

In a review article by Double in (1975) it was suggested that use of xenografts in chemotherapy testing might be as a secondary screen for new agents rather than as a test for individual patient's tumours, but it seemed possible that improvements in technique might have invalidated this prediction. We therefore analysed our data with regard to five tumour types extensively studied at our laboratories to assess whether xenografts could be used for individual patient drug sensitivity testing.

Throughout this study, immunosuppressed CBA/lac mice were used for tumour implantation. The immunosuppression was effected by thymectomy at 4 weeks of age, followed by 9 Gy whole-body irradiation 4–8 weeks later. The mice were protected against the otherwise lethal effect of radiation by an intra-peritoneal injection of $200\,\mathrm{mg\,kg^{-1}}$ of cytosine-arabinoside given 48 h prior to exposure (Steel *et al.*, 1978). Specimens of primary and metastatic tumour were collected at surgery on patients at the Royal Marsden Hospital and other collaborating hospitals. Specimens were placed in transport medium at 4°C and taken to the laboratory for implantation with a minimum possible delay. Only viable specimens from histologically proven tumours were used. Frozen sections were obtained routinely in patients with breast cancer, but not always in other tumour types.

Pieces of tumour between 2 and 5 mm diameter were implanted into s.c. pocket over the flank of each animal within one week of immunosuppression. At least one representative portion was sent for histology to confirm that viable tumour was being implanted. Each mouse was regularly inspected for signs of tumour growth for a period of one year. When progressive growth ensued, the tumour was allowed to reach a size of ~1 cm diameter, after which the mouse was killed and the tumour excised. The tumour was then divided into cubes, and transplanted into as many immunosuppressed mice as tumour bulk permitted (usually 10–20). Several cubes were examined histologically.

Drug testing was carried out using a control group of 5–10 tumour bearing mice, and groups of 5–10 mice with tumours for each drug to be tested. Testing was performed on the earliest passage at which sufficient tumour bearing mice could be produced.

All patients were followed by personal interview and examination for at least one year, or until the patient died. Further details of implantation technique, histological methods and chemosensitivity testing have been published elsewhere (Bailey *et al.*, 1980*b*; Raghaven *et al.*, 1980*a*, 1980*b*; Selby *et al.*, 1979, 1980; Jones *et al.*, in preparation; Shorthouse *et al.*, 1980). Specimens from 339 patients were implanted and shown to

Correspondence: M.J. Bailey, St George's Hospital, Blackshaw Road, London, S.W.17.
Received 8 July 1984; accepted 16 August 1984.

© The Macmillan Press Ltd., 1984

**Table I** Number of serially transplantable xenografts established by tumour type

| *Tumour type* | *Total viable specimens implanted* | *Viable specimens from untreated patients* | *Serially transplantable lines* |
|---|---|---|---|
| Breast carcinoma | 91 | 91 | 9 |
| Testicular teratoma | 46 | 34 | 11 |
| Ovarian carcinoma | 37 | 20 | 7 |
| Bronchogenic carcinoma | 49 | 48 | 38 |
| Melanoma | 16 | 10 | 10 |
| Overall | 239 | 203 | 75 |

**Table II** Clinically usable lines established by tumour type.

| *Tumour type* | *Number of transplantable lines* | *Lines in which donor patients died before testing* | *Lines with low take rate or long doubling time* | *Useful takes* |
|---|---|---|---|---|
| Breast carcinoma | 9 | 0 | 3 | 6 |
| Testicular teratoma | 11 | 2 | 2 | 7 |
| Ovarian carcinoma | 7 | 4 | 1 | 2 |
| Bronchogenic carcinoma | 38 | 29 | 4 | 5 |
| Melanoma | 10 | 5 | 1 | 4 |
| Overall | 75 | 40 | 11 | 24 |

contain viable tumour on histological review. From these implants, 75 serially transplantable xenograft lines have been established, an overall take risk of 31%. The take rate varied considerably from one tumour type to another, the lowest being breast carcinoma (10%), the highest being bronchial carcinoma (77%) (Table I). Of the 239 patients from whom specimens were implanted, 201 had not had previous chemotherapy, although some had received radiotherapy. Certain tumour implants, such as those of melanoma and ovary were sometimes derived from patients with very advanced disease and these patients have been excluded from our final analysis so as not to bias the results by inclusion of patients with a short life expectancy. The 75 xenograft lines produced 24 lines which could have yielded chemosensitivity data prior to the patient's death. Of the remaining lines, some were so slowly growing with such a low take rate after serial passage as to prevent chemotherapy testing. In others the patient had died before such testing could be carried out. Therefore, out of the 239 patients, 24 could have had chemosensitivity testing performed prior to their death (10%). Excluding patients with advanced disease and those who had been previously treated, 11% overall (22/201) could have been tested (Table II). The proportion of usable takes (in terms of drug sensitivity testing) varied from 6.6% for breast carcinoma to 25% for malignant melanomas.

The overall take rate in our series refers to serially transplantable xenograft lines and was 31%. This is very similar to the results obtained by other groups with an extensive experience of human tumour xenografting, whether using nude or immunosuppressed mice with published take rates of between 12 and 26% (Shimosato *et al.*, 1976; Povlsen, 1978; Giovanella *et al.*, 1978).

Work undertaken at our institute has shown no difference in take rates between the immunosuppressed mouse used in these studies and the congenitally athymic mouse often used as an alternative (Steel *et al.*, 1978; Bailey *et al.*, 1980 ). Considerable effort has been directed towards raising the take rate but no dramatic increase in establishing serially transplantable human tumour lines has occurred to suggest that the overall take rate of 31% can be improved.

We have defined a "Usable take" for the purposes of this paper as being a xenograft line established from a human tumour and maintaining histological and karyotypic characteristics of that tumour in serial passage in immunosuppressed mice, which, by virtue of passage, could be implanted into sufficient mice to allow chemotherapy trials to be undertaken yielding results within the lifespan of the donor patient.

We have therefore excluded from our analysis patients whose disease at the time of implantation to the mice was so advanced as to render any prospect of their survival until xenograft testing could be performed unlikely. Even so, only 11% of the patients whose tumours were implanted had xenograft lines established which could have yielded useful drug sensitivity data before their death. It may be that the reason so few patients could have benefited from drug testing is that patients with less aggresive tumours who survived long enough for testing to have been possible had a low xenograft take rate. Table III shows the take rate of each tumour type compared with 3 and 5 year crude survival figures. It can be seen that in general terms, the more lethal the tumour, the more likely it is to be established successfully as a xenograft. The expense involved in drug testing using the xenograft model is considerable – the cost of mice alone, *excluding* feeding, housing, salaries of technicians and clinicians involved would have been £400 per tumour tested for 3 drugs assuming optimal take rates in passaging, and 100% animal survival. (The immunosuppressed mice used in our laboratories are ~25% of the cost of nude mice used in many laboratories, but require more technical expertise to prepare). The cost of testing more than three drugs would be proportionally higher and to be clinically useful, as many as 6–10 drugs would need to be examined. A realistic estimate to include salaries and multiple drug testing, but still excluding capital cost would be in the order of £2000 per tumour tested.

The human tumour xenograft has many applications in cancer research, including the evaluation of new cytotoxic drugs, the study of tumour markers, development of tumour specific antibodies, tumour radiolocalisation, cell kinetics and experimental pathology. However, our results show that even assuming that xenografted tumours maintain the drug sensitivity of the original tumour in the patient, the place of the xenograft model for individual patient drug testing is limited by the small proportion of patients who might benefit and the necessarily long delay between implantation of the surgical specimen and chemotherapy results becoming available. This period was seldom shorter than 30 weeks and usually in excess of 50 weeks. This would be acceptable in patients with breast cancer, who often do not require chemotherapy for recurrent disease for many years, but exceeds the median survival of patients with bronchogenic carcinoma. Therefore, if drug testing results became available during the course of the disease, it would only be at a very late stage. For these reasons, we feel that serially transplantable human tumour xenografts are unlikely to be of value in individual patient drug sensitivity testing.

**Table III** Percentage take rate compared with 3- and 5-year survival rates for the 5 tumour types studied

| *Tumour types* | *Take rate as xenograft* (%) | *Overall survival* 3 *years* (%) | 5 *years* (%) |
|---|---|---|---|
| Breast | 10 | 70 | 50 |
| Ovary | 19 | 40 | 25 |
| Teratoma | 24 | 45 | 40 |
| Melanoma (recurrent) | 63 | 20 | 10 |
| Bronchus | 75 | 6 | 4 |

### References

BAILEY, M.J., GAZET, J.-C. & PECKHAM, M.J. (1980*a*). Human breast carcinoma xenografts in immunosuppressed mice. *Br. J. Cancer*, **42**, 000.

BAILEY, M.J., GAZET, J.-C., SMITH, I.E. & STEEL, G.G. (1980*b*). Chemotherapy of human breast carcinoma xenografts. *Br. J. Cancer*, **42**, 524.

CASTRO, J.E. (1972). Human tumours grown in mice. *Nature*, (New Biol.), **239**, 83.

DOUBLE, J.A. (1975). Human tumour xenografts. *Biomedicine*, **22**, 461.

GIOVANELLA, B.C., STEHLIN, J.S., WILLIAMS, L.J., LEE, S.S. & SHEPARD, R.C. (1978). Heterotransplantation of human cancers into nude mice. *Cancer*, **42**, 2269.

HAYAHASHI, H., KAMEYA, T., SHIMOSATO, Y. & MUKOJIMA, T. (1978). Chemotherapy of human choriocarcinoma transplanted into nude mice. *Am. J. Obstet. Gynaecol.*, **131**, 548.

NOWAK, K., PECKHAM, M.J. & STEEL, G.G. (1978). Variations in response of xenografts of colo-rectal carcinoma to chemotherapy. *Br. J. Cancer*, **37**, 576.

POVLSEN, C.O., SPANG-THOMPSEN, M., RYGAARD, J. & VISFELDT, J. (1978). Heterotransplantation of human malignant tumours to athymic nude mice. In: *Immunodeficient Animals for Cancer Research*. (Ed. Sparrow), McMillan Press, p. 00.

POVLSEN, C.O. & RYGAARD, J. (1974). Effects of cyclophosphamide on a Burkitt's lymphoma serially grown in nude mice. In *Proceedings of the First International Workshop on Nude Mice*. (Eds. Rygaard & Povlsen). Stuttgard: C.F. Verlag, p. 285.

POVLSEN, C.O. (1978). Aspects of Treatment of Human Cancer in the Nude Mouse. In *Experimental and Clinical Research*. (Eds. Fogh & Giovanella), New York: Academic Press, p. 450.

RAGHAVEN, D., GIBBS, J., NEVILLE, A.M. & PECKHAM, M.J. (1980*a*). Experimental germ cell tumours. *N. Engl. J. Med.*, **302**, 811.

RAGHAVEN, D., GIBBS, J., COSTA, R.N. & 4 others. (1980*b*). The interpretation of marker protein assays: A critical appraisal in clinical studies and a xenograft model. *Br. J. Cancer*, **42**, (Suppl. IV), 191.

RYGAARD, J. & POVLSEN, C.O. (1969). Heterotransplantation of a human malignant tumour to a "nude" mouse. *Acta Pathol. Microbiol. Scand.*, **77**, 758.

SELBY, P.J., THOMAS, J.M., MONAGHAN, P., SLOANE, J. & PECKHAM, M.J. (1980). Human tumour xenografts established and serially transplanted in mice immunologically deprived by thymectomy, cytosine arabinoside and whole-body irradiation. *Br. J. Cancer*, **41**, 52.

SELBY, P.J., HEYDERMAN, E., GIBBS, J. & PECKHAM, M.J. (1979). A human teratoma serially transplanted in immuno-deprived mice. *Br. J. Cancer*, **39**, 578.

SHIMOSATO, Y., KAMENA, T., NAGAI, K. & 4 others. (1976). Transplantation of human tumours in nude mice. *J. Natl Cancer Inst.*, **56**, 1251.

SHORTHOUSE, A.J., PECKHAM, M.J., SMYTH, J.F. & STEEL, G.G. (1980). The therapeutic response of bronchial carcinoma xenografts in a direct patient xenograft comparison. *Br. J. Cancer*, (Suppl. IV), 142.

STEEL, G.G., COURTENAY, V.D. & ROSTOM, A.Y. (1978). Improved immuno-suppression techniques for the xenografting of human tumours. *Br. J. Cancer*, **37**, 224.